# Common genetic variants in *ADCY5* and gestational glycemic traits

**DOI:** 10.1371/journal.pone.0230032

**Published:** 2020-03-12

**Authors:** Rong Lin, Ziyu Yuan, Caicai Zhang, Hongfang Ju, Yuantian Sun, Na Huang, Langxin Chen, Li Jin

**Affiliations:** 1 Department of Biology, Hainan Medical University, Haikou, Hainan, China; 2 State Key Laboratory of Genetic Engineering, and Collaborative Innovation Center for Genetics and Development, School of Life Sciences, Fudan University, Shanghai, China; 3 Fudan University Taizhou Institute of Health Sciences, Taizhou, Jiangsu, China; 4 Department of Physiology, Hainan Medical University, Haikou, Hainan, China; 5 Department of Gynaecology and Obstetrics, Taizhou People's Hospital, Taizhou, Jiangsu, China; 6 CAS-MPG Partner Institute for Computational Biology, Shanghai Institute for Biological Sciences, Chinese Academy of Sciences, Shanghai, China; University of Cambridge, UNITED KINGDOM

## Abstract

Two meta-analysis of genome wide association studies identified two variants at adenylate cyclase 5 (*ADCY5*) associated with type 2 diabetes mellitus, fasting and 2-hour glucose in non-pregnant individuals of European descent. The objective of our study was to explore the role of common variants in *ADCY5* on gestational glycemic traits, including plasma glucose, insulin values, β cell function and insulin resistance in the fasted state as well as plasma glucose 1 hour after a 50-gram glucose challenge test among Chinese Han women. Homoeostasis model assessment (HOMA) was used to quantify β cell function (HOMA1-β and HOMA2-β) and insulin resistance (HOMA1-IR and HOMA2-IR). Thirty-five single nucleotide polymorphisms (SNPs) in *ADCY5* were genotyped in 929 unrelated Chinese Han women with singleton pregnancies. Three SNPs (rs6797915, rs9856662 and rs9875803) displayed evidence for association with plasma glucose 1 hour after a 50-gram glucose challenge test (*P* = 0.042, 0.018 and 0.018, respectively), one (rs6777397) displayed evidence for association with HOMA1-β (*P* = 0.014), and one (rs6762009) displayed evidence for association with HOMA1-IR (*P* = 0.033). These results provide additional insight into the effects of genetic variation within *ADCY5* in glucose metabolism, especially during pregnancy and in non-European descent populations.

## Introduction

Gestational glycemic traits and gestational diabetes mellitus (GDM) are usually the results of the interplay between environmental and genetic factors [1, 2]. Published heritability estimates for fasting plasma glucose (FPG) levels during pregnancy range between 30% and 71% [1]. Adverse pregnancy outcomes increase with increasing plasma glucose levels during pregnancy [3]. These increases are continuous throughout the range of plasma glucose levels, even among those levels under the cut-off points for the diagnosis of GDM. So it is difficult to ascertain a specific level at which elevated glucose levels become clinically significant. An international consensus could not be reached on the optimal diagnostic cut-off points for GDM and thereby diagnostic criteria for GDM used in studies are different [4]. Furthermore, the intermediate phenotypes of GDM may provide more power to detect genetic variants compared with the more distal clinical diagnosis. Therefore, in comparison with case-control studies of GDM, description of the effects of genetic variants on gestational glycemic traits, such as FPG and insulin sensitivity, may be better.

Adenylate cyclase 5 (ADCY5) is a member of the membrane-bound adenylate cyclase (ADCY) family, which converts adenosine triphosphate (ATP) to the second messenger cyclic adenosine monophosphate (cAMP) and pyrophosphate [5]. cAMP itself is a key regulator for glucose and lipid metabolism [6, 7]. cAMP can regulate glucose-induced insulin secretion through several possible mechanisms [7, 8]. ADCY5 may be the most abundant member of the ADCY family in human islets [9, 10]. ADCY5 couples glucose to insulin secretion by converting glucose signals into cAMP production [11]. Genome wide association studies (GWASs) have revealed variants at *ADCY5* associated with type 2 diabetes mellitus (T2DM), fasting and 2-hour glucose [12, 13]. The genetic etiology of GDM has been documented to overlap that of T2DM [2]. However, knowledge regarding the genetic risk variants in *ADCY5* for GDM and glycemic traits in pregnancy is limited, although two studies have found an association between *ADCY5* variant and GDM [14, 15]. It remains unclear whether *ADCY5* variants are associated with glycemic traits in pregnancy. We therefore tested the hypothesis.

In this study, we conducted a cross-sectional study to assess the influence of common variants in *ADCY5* on gestational glycemic traits, including fasting plasma glucose and insulin, β cell function, insulin resistance as well as plasma glucose 1 hour after a 50-gram glucose challenge test among pregnant women. Further, for better evaluation, 35 tagging single nucleotide polymorphisms (SNPs) were selected to cover the known common variants of *ADCY5*.

## Materials and methods

### Study population

This study enrolled 929 unrelated Chinese Han women with singleton pregnancies from Taizhou People's Hospital between 2010–2013. The participants were aged between 19 and 42 (mean: 26.8±3.8) years. Most of the antenatal check-ups for all participants were performed in Taizhou People's Hospital. A screening test (50-gram 1-hour glucose challenge test) for GDM after overnight fasting of 8 to14 hours was offered to all participants around 24–28 weeks’ gestation. Fasting glucose and insulin levels were also measured at the same time. For those participants whose FPG ≥5.6 mmol/l or plasma glucose 1 hour after a 50-gram glucose challenge test ≥7.8 mmol/l, a diagnostic two-hour, 75-gram oral glucose tolerance test (OGTT) was conducted within one week. Plasma glucose was detected using a hexokinase reaction on a Cobas P800 analyzer (Roche Diagnostics GmbH, Mannheim, Germany). Plasma insulin was determined by an electrochemiluminescence assay on a Cobas e601 platform (Roche Diagnostics GmbH, Mannheim, Germany). FPG levels of almost 94.4% of participants were lower than 5.6 mmol/l. Women who reportedly received treatment against diabetes before the screening test were not included. To exclude the effects of assisted reproduction, this study only enrolled women who naturally conceived. In order to avoid sampling errors, all eligible pregnant women were invited, regardless of age and occupation. As long as they were willing to participate, they would be collected.

Homoeostasis model assessment (HOMA) was used to estimate basal β cell function and insulin resistance. HOMA1 β cell function (HOMA1-β) index was defined as (20×fasting insulin)/(fasting glucose-3.5). HOMA1 insulin resistance (HOMA1-IR) index was calculated as fasting insulin×fasting glucose/22.5. When FPG levels are ≤3.5 mmol/l, HOMA1-β can not be estimated and thereby HOMA1-β values of 20 subjects were unavailable. HOMA2-β and HOMA2-IR indices were estimated with the HOMA2 calculator version 2.2 released by the Diabetes Trials Unit at the Oxford Centre for Diabetes, Endocrinology and Metabolism, University of Oxford (*if Glucose: 3.0 to 25.0 mmol/l and Insulin: 20 to 400 pmol/l) [16, 17]. HOMA2-β and HOMA2-IR indices of 844 participants were obtainable. According to Wallace *et al*. [17], HOMA2 more accurately indicates the metabolic process because it models the feedback relation between insulin and glucose in various organs of the human body. HOMA1-IR primarily manifests hepatic insulin resistance, and HOMA2-IR manifests hepatic and peripheral insulin resistance [17–19].

Genomic DNA was isolated from venous blood samples which were drawn from the participants in the days surrounding the delivery. Ethical approval was provided by the Ethics Committee of Hainan Medical University and Fudan University Taizhou Institute of Health Sciences. All participants gave written, informed consent. Basic characteristics of all participants are shown in Table 1. Fifty-one participants had missing fasting insulin data and forty-eight had missing 50-g 1-h glucose data.

**Table 1:** Basic characteristics of study participants.

Characteristics	N	
Age at childbirth, years	926	26.8±3.8
Spouse's age at childbirth, years	923	28.2±4.4
Neonatal sex, % male	927	52.90%
Prepregnancy gravidity	927	0.7±1.0
Prepregnancy parity	927	0.2±0.4
Prepregnancy BMI, kg/m^2^	915	20.6±2.6
24-28-week fasting plasma glucose, mmol/l	929	4.54±0.62
24-28-week fasting plasma insulin, pmol/l	878	63.52±73.30
24-28-week 1-hour plasma glucose after a 50-gram glucose challenge test, mmol/l	881	7.13±1.45
HOMA1-β	858	226.54±253.05
HOMA1-IR	858	1.90±2.58
HOMA2-β	844	126.95±45.20
HOMA2-IR	844	1.09±0.58

Data are expressed as arithmetic mean±standard deviation or percentages. BMI: body mass index; HOMA-β: homeostasis model assessment-β-cell function; HOMA-IR: homeostasis model assessment-insulin resistance.

### SNP genotyping

Thirty-five tag SNPs across the *ADCY5* gene (chromosome 3: 123,282,296..123,459,758 177.46 kbp, human genome reference assembly GRCh38/hg38), including rs6801826, rs6777397, rs10049128, rs9857526, rs10934643, rs4678005, rs12633873, rs6770805, rs9870651, rs4234214, rs13058985, rs6806851, rs4678008, rs4678010, rs4677884, rs4450740, rs4596093, rs6795648, rs2332510, rs11923120, rs6797915, rs12496583, rs6794936, rs9856662, rs12486065, rs7616545, rs7641344, rs9875803, rs6774571, rs6762009, rs4677889, rs9841477, rs4678030, rs13072153 and rs2046487, were identified according to linkage disequilibrium (LD) patterns in the phase III Han Chinese in Beijing (CHB) and Southern Han Chinese (CHS) populations with *r*^2^ thresholds of 0.90 and minor allele frequency (MAF) thresholds of 0.05 (data derived from the 1000 Genomes Project, based on GRCh38). A list of the included SNPs is provided in S1 Table.

The genotypes of all SNPs were determined by SNPscan^TM^ technology (Genesky Biotechnologies Inc., Shanghai, China), which was based upon double ligation and multiplex fluorescence polymerase chain reactions. For quality control purpose, we randomly selected 5.5% of the samples and performed genotyping twice independently, which yielded >99% concordance rates. In 99.9% or more of the samples, each polymorphism was successfully genotyped (S1 Table). All 35 SNPs genotyped were in Hardy-Weinberg equilibrium (HWE) with *P*>0.05.

### Statistical analysis

HWE was tested for each polymorphism separately using a χ^2^ test. The strength of pairwise LD between the 35 variants was measured as *r*^2^ using the online platforms SHEsis (http://analysis.bio-x.cn/myAnalysis.php) and SNPStats (http://bioinfo.iconcologia.net/snpstats/start.htm). Pairwise variants exhibit low LD (*r*^2^<0.50), moderate high LD (0.80>*r*^2^>0.50), high LD (*r*^2^>0.80) and perfect LD (*r*^2^ = 1). If *r*^2^ between two variants is <0.80, they were thought to be independent.

The impact of the 35 SNPs on glycemic traits including 24-28-week FPG, plasma glucose 1 hour after a 50-gram glucose challenge test, fasting insulin, HOMA1-β, HOMA1-IR, HOMA2-β and HOMA2-IR, were assessed using analysis of variance (ANOVA). Analysis of covariance (ANCOVA) was carried out for adjustment of age at childbirth, neonatal sex, prepregnancy gravidity and prepregnancy parity. Fasting insulin levels, HOMA1-β, HOMA1-IR, HOMA2-β and HOMA2-IR were natural log transformed to approximate normality before ANOVA and ANCOVA. In each analysis, cases with missing data were simply dropped and then the remaining data were analyzed. Statistical analyses were done using Statistical Package for Social Science (SPSS) version 15.0 (Chicago, IL, USA). In the initial analyses, *P*<0.05 were considered statistically significant. To account for multiple testing, Bonferroni correction was applied.

## Results

**Fig 1:**
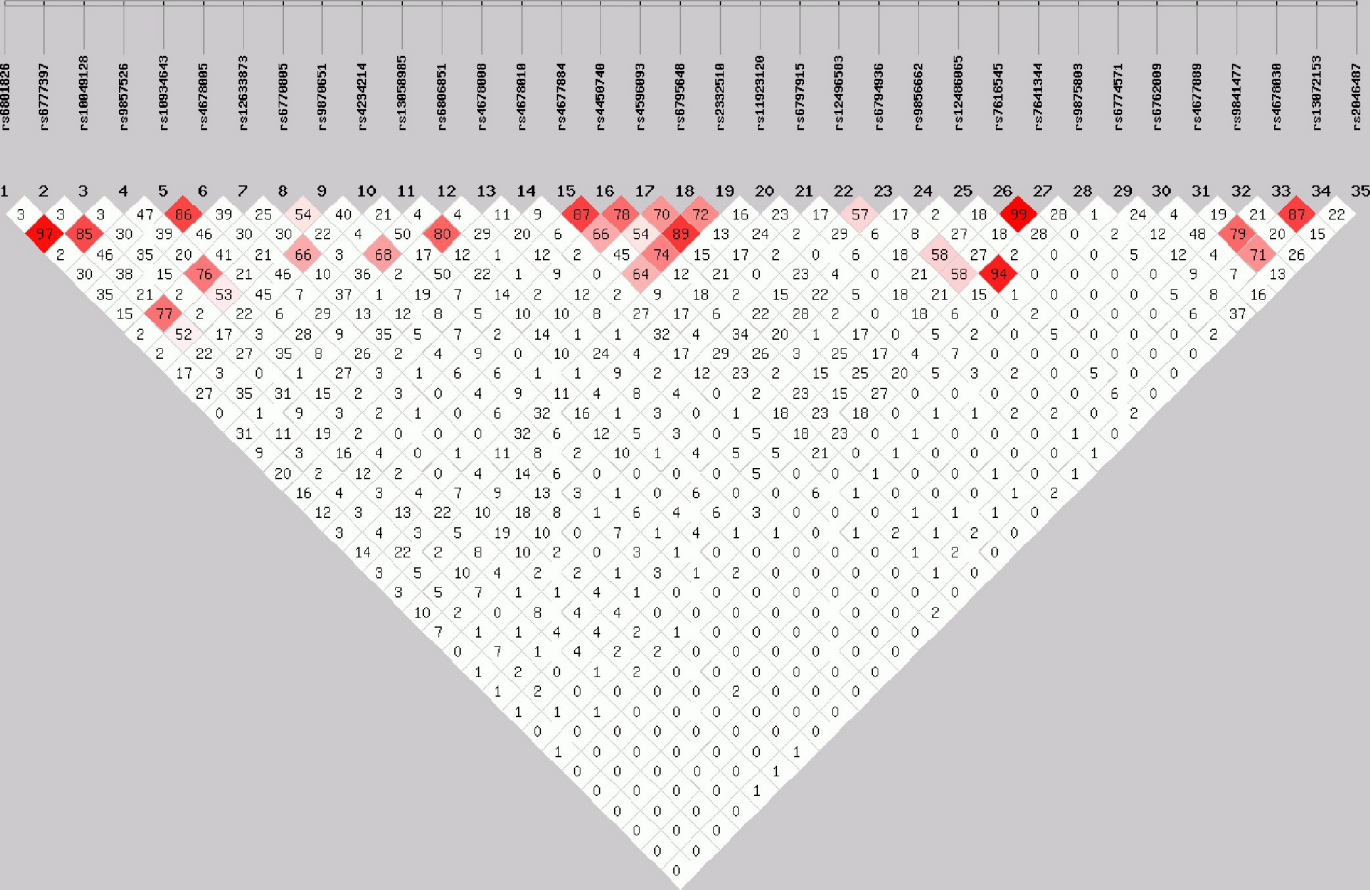
Pairwise linkage disequilibrium between SNPs, as measured by *r*^2^, in the *ADCY5* gene. Numbers within diamonds indicate the *r*^2^ value between the two SNPs defined by the upper left and the upper right sides of the diamond. For example, *r*^2^ between rs6801826 (SNP 1) and rs6777397 (SNP 2) is 0.03. The plot was generated by the SHEsis Online Version. Key: *r*^2^ = 0 is given in white, and 0 < *r*^2^ ≤ 1 is given in shades of red (redder color represents higher *r*^2^).

The *r*^2^ values of 9 SNP pairs (i.e. SNP 1 and 3, SNP 2 and 4, SNP 5 and 6, SNP 11 and 13, SNP 15 and 16, SNP 17 and 19, SNP 24 and 28, SNP 26 and 27, as well as SNP 33 and 34) were all above 0.80, which showed high LD between them (Fig 1). The effective number of independent variants was considered for Bonferroni correction. Accordingly, the corrected significant *P* value was arbitrarily set to 0.002 (i.e. 0.05/26) for *ADCY5* for it corresponds to an adjusted *P* value of 0.05 divided by the number of independent SNPs (26) in the 35 SNPs. We next analyzed the associations between the 35 SNPs and gestational glycemic traits. For brevity, only the results of statistical tests as *P*<0.05 are indicated in Table 2. As shown, five statistically significant associations were found.

**Table 2:** Association between *ADCY5* common variants and gestational glycemic traits.

		Genotype	N	Mean(95% confidence interval)	*P*	*P*^a^
24-28-week 1-hour plasma glucose after a 50-gram glucose challenge test, mmol/l						
SNP 21	rs6797915	GG	355	7.10(6.96–7.24)	0.042	0.067
		GC	407	7.07(6.93–7.21)		
		CC	118	7.44(7.13–7.76)		
SNP 24	rs9856662	CC	681	7.20(7.09–7.32)	0.018	0.019
		CA	186	6.88(6.68–7.08)		
		AA	14	6.78(6.09–7.47)		
SNP 28	rs9875803	TT	675	7.21(7.10–7.32)	0.018	0.019
		TC	191	6.88(6.69–7.08)		
		CC	15	6.84(6.20–7.48)		
HOMA1-β						
SNP 2	rs6777397	GG	574	165.17(156.05–174.82)	0.014	0.022
		GA	249	189.04(173.11–206.44)		
		AA	35	145.21(117.71–179.14)		
HOMA1-IR						
SNP 30	rs6762009	GG	270	1.53(1.42–1.64)	0.033	0.044
		GC	418	1.57(1.48–1.66)		
		CC	169	1.36(1.23–1.50)		

Values are reported as unadjusted arithmetic mean (95% confidence interval) for plasma glucose and geometric mean (95% confidence interval) for other variables as well as only for variants meeting significance criteria (*P*<0.05) in analysis of variance.

^a^ Adjusted with analysis of covariance for age at childbirth, neonatal sex, prepregnancy gravidity and prepregnancy parity.

Significant differences were observed in 24-28-week plasma glucose 1 hour after a 50-gram glucose challenge test among the genotype groups of three SNPs (rs6797915, rs9856662 and rs9875803). Plasma glucose 1 hour after a 50-gram glucose challenge test was significantly higher in homozygous carriers of the minor allele C of SNP rs6797915 compared with carriers of the major allele G (*P* = 0.042). It was also significantly increased in homozygous carriers of the major allele of SNP rs9856662 or rs9875803 as compared to carriers of the corresponding minor allele (*P* = 0.018).

One SNP (rs6777397) was related to HOMA1-β (*P* = 0.014): women carrying heterozygous genotype GA of SNP rs6777397 had significantly higher HOMA1-β as compared to those carrying homozygous genotype GG or AA.

One SNP (rs6762009) was correlated with HOMA1-IR (*P* = 0.033): HOMA1-IR in women homozygous for the minor allele C was significantly lower than that in those with the major allele G.

When adjusted for age at childbirth, neonatal sex, prepregnancy gravidity and prepregnancy parity, the effect of rs6797915 was no longer significant whereas those of rs9856662, rs9875803, rs6777397 and rs6762009 remained significant. There were no significant differences between the 35 SNPs and 24-28-week FPG, fasting insulin, HOMA2-β as well as HOMA2-IR. No significant associations were detected at the 0.002 level. In other words, none of these associations passed Bonferroni correction.

## Discussion

In the present study, we examined whether 35 *ADCY5* common variants were related to glycemic traits during pregnancy, including FPG, plasma glucose 1 hour after a 50-gram glucose challenge test, fasting plasma insulin, HOMA1-β, HOMA2-β, HOMA1-IR, and HOMA2-IR in Chinese Han women. Five associations were observed at *P* <0.05 but none of them would withstand Bonferroni correction for multiple comparisons with a *P* value threshold of 0.002 for statistical significance.

In 2010, two parallel meta-analysis of GWASs in nongravid individuals of European descent identified variants at *ADCY5* (SNP rs11708067 and rs2877716) associated with fasting and 2-hour glucose as well as T2DM [12, 13]. SNP rs11708067 and rs2877716 are in high LD (*r*^2^ = 0.82). These results suggest that *ADCY5* may be involved in glucose metabolism.

Since then, a number of studies have attempted to replicate the associations of *ADCY5* variants with glycemic traits and/or T2DM. However, most of these studies were conducted in populations of European descent and reported positive results [20–24]. A study, which was performed in a population consisting of 56.4% of European descent and 43.6% of non-European descent, also got positive results [25]. Three studies were conducted in Asians, two in Chinese Han [26, 27] and one in South Asians of Punjabi ancestry [28]. Of note, most studies investigated only one or two *ADCY5* variants (i.e. SNP rs11708067 and/or rs2877716) [20–26, 28]. The effects of other *ADCY5* variants on glycemic traits, especially in populations of non-European ancestry, remained largely unknown. Our study analyzed 35 *ADCY5* SNPs in a Chinese Han population.

Hu *et al*. (2010) [26] investigated only two *ADCY5* variants (SNP rs11708067 and rs2877716) and Sun *et al*. (2015) [27] investigated SNP rs11708067 and rs9883204. SNP rs9883204 is in perfect LD with rs2877716 in many populations such as CHB, Utah residents of northern and western European ancestry from the CEPH collection (CEU) (*r*^2^ = 1) (data derived from the 1000 Genomes Project, based on GRCh38). These two studies were both conducted in Chinese Han and failed to replicate the associations with the risk of T2DM or impaired glucose metabolism and glycemic traits. SNP rs11708067, rs2877716 and rs9883204 are much rarer in Chinese Han (MAF 0.002–0.005) than they are in the European ancestry populations (MAF 0.130–0.250). There might be insufficient statistical power to replicate the effects of the three variants because of their rarity. It is unclear whether there are other *ADCY5* variants with effects on glucose metabolism in Chinese Han. Therefore, the present study included another 35 SNPs other than the three variants.

In addition to SNP rs11708067 and rs9883204, Sun *et al*. (2015) [27] also investigated SNP rs6777397 (SNP 2), rs9881942, rs4678017 and rs7641344 (SNP 27). In CHB and CHS populations, SNP rs9881942 and rs4678017 were in high LD with rs2332510 (SNP 19) and rs11923120 (SNP 20), respectively (both *r*^2^>0.976) (data derived from the 1000 Genomes Project, based on GRCh38). They only observed that SNP 27 was associated with HOMA1-β (*P* = 0.02) and fasting insulin levels (*P* = 0.05). Note that they did not apply multiple testing corrections. In the present study, SNP 27 was not related to any gestational glycemic traits investigated even before multiple testing corrections. Among SNP 2, 19, 20 and 27, the present study only showed SNP 2 was associated with HOMA1-β but the association did not pass Bonferroni correction. Discrepancies between the studies may be due to the facts that (1) the indexes of glucose metabolism in the study of Sun *et al*. were measured during non-pregnancy while the indexes in the present study were measured during pregnancy, and (2) only 52% of the participants in the study of Sun *et al*. were female while the participants in the present study were all female.

To our knowledge, until now, there have been only two studies on the relationship between *ADCY5* genetic variants and glucose metabolism in pregnant women [14, 15]. Huopio *et al*. (2013) [14] observed that SNP rs11708067 was associated with GDM and OGTT 2-hour glucose level in Finnish pregnant women. Arora *et al*. (2018) [15] also found that SNP rs11708067 was associated with GDM in South Asian pregnant women of Punjabi ancestry. Both studies focused on only one genetic variant of the *ADCY5* gene (i.e., SNP rs11708067) which is rare in Chinese Han.

Most studies in non-pregnant European populations have consistently shown that *ADCY5* genetic variants were related to glycemic traits and/or T2DM, and two studies have reported that SNP rs11708067, a genetic variant of *ADCY5*, was associated with GDM. Functional studies also showed that ADCY5 is involved in glucose metabolism. To date, genetic studies of *ADCY5* were mainly conducted in non-pregnant European populations, but less in non-European populations and pregnant populations. Genetic variants of *ADCY5* have large allele frequency difference among different races. Therefore, it is necessary to confirm the relationship between genetic variants of *ADCY5* and glucose metabolism particularly in non-European populations and pregnant populations.

Due to both differential LD structure between populations and differences in SNP coverage, the present study assessed the relationship between 35 *ADCY5* SNPs and glycemic traits in a Chinese Han pregnant population and identified five statistical significant associations. However, the five associations were weak, i.e., with *P* values ranging from 0.042 to 0.014, and none of them could withstand Bonferroni correction for multiple comparisons. This is a limitation of the study. Another limitation is the lack of functional evidence. The five statistical significant SNPs have not yet been identified as true causal variants. Therefore, the current findings should be considered preliminary and need to be validated by further investigations using large replication samples.

## Conclusions

In summary, the present study comprehensively evaluated the genetic associations of common variants in *ADCY5* with gestational glycemic traits in a Chinese Han population and identified five variants associated with gestational glycemic traits before Bonferroni correction. Following Bonferroni correction, these associations became insignificant. Therefore, these nominally significant associations still need to be verified in other populations. Our findings expanded the body of knowledge about the effects of *ADCY5* on glycemic traits, especially in non-European descent populations and during pregnancy, although the effects need to be addressed in future studies.

## Supporting information

S1 Table Primary information for genotyped SNPs. (DOC)
