# Supplementary material for: Common genetic variants in ADCY5 and gestational glycemic traits
Source: PLoS One. 2020 Mar 12;15(3):e0230032. doi: 10.1371/journal.pone.0230032 (PMC7067392; doi:10.1371/journal.pone.0230032)
Supplement: S1 Table — (DOC) [file pone.0230032.s001.doc]

| **S1 Table. Primary information for genotyped SNPs.** | | | | | | | |
| --- | --- | --- | --- | --- | --- | --- | --- |
| **No.** | **dbSNP ID** | **Chromosome position^a^** | **Minor/Major allele** | **Location in gene region** | **Call rate** | **HWE (*P* value)** | **MAF** |
|
| 1 | rs6801826 | Chr 3:123287551 | C/G | intron | 1.000 | 0.213 | 0.128 |
| 2 | rs6777397 | Chr 3:123287606 | A/G | intron | 1.000 | 0.417 | 0.184 |
| 3 | rs10049128 | Chr 3:123292514 | T/C | intron | 1.000 | 0.280 | 0.125 |
| 4 | rs9857526 | Chr 3:123293798 | G/A | intron | 0.999 | 0.895 | 0.182 |
| 5 | rs10934643 | Chr 3:123298186 | C/T | intron | 1.000 | 0.096 | 0.320 |
| 6 | rs4678005 | Chr 3:123302451 | T/C | intron | 1.000 | 0.257 | 0.290 |
| 7 | rs12633873 | Chr 3:123308978 | C/T | intron | 0.999 | 0.174 | 0.464 |
| 8 | rs6770805 | Chr 3:123310026 | G/A | intron | 1.000 | 0.389 | 0.186 |
| 9 | rs9870651 | Chr 3:123313968 | T/G | intron | 1.000 | 0.773 | 0.216 |
| 10 | rs4234214 | Chr 3:123314846 | C/T | intron | 1.000 | 0.512 | 0.405 |
| 11 | rs13058985 | Chr 3:123321481 | G/C | intron | 1.000 | 0.769 | 0.135 |
| 12 | rs6806851 | Chr 3:123324064 | T/C | intron | 1.000 | 0.766 | 0.259 |
| 13 | rs4678008 | Chr 3:123326561 | T/C | intron | 1.000 | 0.371 | 0.119 |
| 14 | rs4678010 | Chr 3:123337306 | C/T | intron | 1.000 | 0.986 | 0.474 |
| 15 | rs4677884 | Chr 3:123344123 | C/G | intron | 1.000 | 0.054 | 0.288 |
| 16 | rs4450740 | Chr 3:123349262 | T/C | intron | 1.000 | 0.120 | 0.271 |
| 17 | rs4596093 | Chr 3:123349439 | C/T | intron | 0.999 | 0.142 | 0.320 |
| 18 | rs6795648 | Chr 3:123370219 | A/G | intron | 1.000 | 0.638 | 0.258 |
| 19 | rs2332510 | Chr 3:123377475 | G/C | intron | 1.000 | 0.078 | 0.307 |
| 20 | rs11923120 | Chr 3:123382745 | A/G | intron | 0.999 | 0.364 | 0.293 |
| 21 | rs6797915 | Chr 3:123386938 | C/G | intron | 0.999 | 0.848 | 0.362 |
| 22 | rs12496583 | Chr 3:123402439 | G/A | intron | 1.000 | 0.062 | 0.302 |
| 23 | rs6794936 | Chr 3:123403467 | T/A | intron | 0.999 | 0.175 | 0.429 |
| 24 | rs9856662 | Chr 3:123411049 | A/C | intron | 1.000 | 0.907 | 0.125 |
| 25 | rs12486065 | Chr 3:123413917 | A/T | intron | 1.000 | 0.449 | 0.150 |
| 26 | rs7616545 | Chr 3:123415963 | G/A | intron | 1.000 | 0.067 | 0.335 |
| 27 | rs7641344 | Chr 3:123416397 | A/G | intron | 0.999 | 0.080 | 0.335 |
| 28 | rs9875803 | Chr 3:123416602 | C/T | intron | 1.000 | 0.914 | 0.130 |
| 29 | rs6774571 | Chr 3:123420037 | T/A | intron | 0.999 | 0.960 | 0.298 |
| 30 | rs6762009 | Chr 3:123420187 | C/G | intron | 0.999 | 0.993 | 0.438 |
| 31 | rs4677889 | Chr 3:123424425 | G/A | intron | 0.999 | 0.618 | 0.292 |
| 32 | rs9841477 | Chr 3:123438630 | A/C | intron | 1.000 | 0.975 | 0.336 |
| 33 | rs4678030 | Chr 3:123457900 | G/A | 5’upstream | 1.000 | 0.766 | 0.316 |
| 34 | rs13072153 | Chr 3:123459285 | G/A | 5’upstream | 1.000 | 0.612 | 0.290 |
| 35 | rs2046487 | Chr 3:123459471 | G/A | 5’upstream | 1.000 | 0.494 | 0.376 |
| SNP: single nucleotide polymorphism; HWE: Hardy–Weinberg equilibrium; MAF: minor allele frequency.  ^a^Positions according to the human genome assembly Build 38. | | | | | | | |
